# Association of psychological resilience with all-cause and cause-specific mortality in older adults: a cohort study

**DOI:** 10.1186/s12889-024-19558-8

**Published:** 2024-07-25

**Authors:** Xiang Wang, Wei Jie, Xionghong Huang, Feng Yang, Yueting Qian, Ting Yang, Miao Dai

**Affiliations:** 1 Jiujiang CityKey Laboratory of Cell Therapy, Jiujiang NO.1 People’s Hospital, Jiujiang, Jiangxi 332000 China; 2 Department of Cardiology, Jiujiang NO.1 People’s Hospital, Jiujiang, Jiangxi 332000 China; 3 Department of Geriatrics, Jiujiang NO.1 People’s Hospital, Jiujiang, Jiangxi 332000 China; 4 Chronic Disease Management Center, Jiujiang NO.1 People’s Hospital, Jiujiang, Jiangxi 332000 China

**Keywords:** Psychological resilience, Older adults, Mortality, Cohort study

## Abstract

**Background:**

Psychological resilience has been associated with increased longevity in the oldest old; however, its significance in the broader older adult population has not been thoroughly explored. There is a lack of understanding regarding its relationship with cause-specific mortality in older adults. This study aims to address these gaps by investigating the association between psychological resilience and both overall mortality and cause-specific mortality in individuals aged 65 and older.

**Methods:**

We enrolled 4,935 participants aged 65 and older in the Chinese Longitudinal Healthy Longevity Survey, with baseline assessments conducted in 2014 and follow-up surveys in 2018. To evaluate the associations between psychological resilience and mortality, we used Cox proportional hazards models. Additionally, we employed restricted cubic spline plots to illustrate the dose-response relationships between these variables.

**Results:**

During a mean (Standard Deviation) follow-up of 3.2 years (1.2), 1726 participants died. Higher psychological resilience was independently associated with lower all-cause mortality risk (Hazard ratio [HR] 0.74, 95% confidence interval [CI]: 0.67–0.82) and cause-specific mortality from cardiovascular disease (HR 0.74, 95% CI: 0.59–0.93), respiratory diseases (HR 0.63, 95% CI:0.45–0.87), and other causes (HR 0.69, 95% CI: 0.60–0.78), excluding cancer-related mortality. Similar effects were evident when examining the psychological resilience score. The dose-response analysis further indicated a gradual decrease in mortality risk corresponding to higher psychological resilience scores. Interaction analyses revealed that psychological resilience has a more pronounced effect on mortality from other causes among economically independent older adults (P-interaction = 0.02).

**Conclusions:**

Enhanced psychological resilience is independently associated with reduced all-cause and some cause-specific mortality in older adults. These findings underscore the importance of addressing psychological factors in the promotion of healthy aging and longevity.

**Supplementary Information:**

The online version contains supplementary material available at 10.1186/s12889-024-19558-8.

## Background

The global demographic shift towards an aging population emerges as one of the most significant trends of the 21st century. As of 2019, the worldwide population of older adults was approximately 700 million [1]. Projections indicate that this number will double by 2050, with the population of individuals aged 80 and above expected to triple during the same period [1]. This demographic transition presents various opportunities and challenges for societies worldwide. One of the primary challenges is the escalating burden of chronic diseases and age-related health conditions, which significantly heighten the mortality risk among older adults. Confronting this demographic reality underscores the necessity to investigate the multifaceted determinants of mortality in older adults, informing strategies aimed at promoting healthy aging and extending life expectancy.

The relationship between psychological resilience and mortality risk has been an area of growing interest, holding significant implications for public health interventions targeting the well-being and longevity of aging populations. Psychological resilience is defined as an individual’s ability to adapt and rebound from adversity, stress, and challenging life events [2, 3]. Previous two studies in China explored the relationship between psychological resilience and longevity among centenarians and older adults aged ≥ 80 years [4, 5], respectively, illuminating the inverse association between higher psychological resilience and all-cause mortality, emphasizing the potential role of psychological factors in promoting longevity. The association between psychological resilience and mortality risk is grounded in several theoretical frameworks and mechanisms. Psychological resilience may mitigate the detrimental effects of stress on health. Resilient individuals often exhibit better stress management skills, leading to lower levels of stress hormones such as cortisol [6]. By buffering the physiological stress response, resilience can reduce the risk of these stress-related diseases. Resilient individuals are more likely to engage in health-promoting behaviors such as regular physical activity, a balanced diet, and adherence to medical advice [7]. These behaviors are critical in preventing and managing chronic diseases, thus potentially lowering mortality rates [8]. Psychological resilience is often associated with stronger social support networks, which provide emotional and practical assistance during health crises [9]. Resilient individuals tend to exhibit better immune responses, including the production of anti-inflammatory cytokines, which can lower the risk of infections and improve disease management [10]. While previous research has established an inverse relationship between psychological resilience and all-cause mortality [4, 5], the extent to which this relationship varies by specific causes of death remains unclear. By distinguishing between different causes of mortality, we can identify particular health conditions that are more influenced by psychological resilience, thereby offering a nuanced understanding of its protective effects. In addition, previous studies predominantly focused on this specific age group. The broader applicability of the finding to the general older adult population remains a research gap that warrants exploration.

This study aims to bridge existing gaps in the literature by conducting a comprehensive cohort study that investigates the association between psychological resilience and both all-cause and cause-specific mortality in older adults, providing valuable insights into the mechanisms underlying healthy aging and longevity in this growing segment of the population.

## Methods

### Study design and participants

The study employed a prospective cohort design utilizing data from the Chinese Longitudinal Healthy Longevity Survey (CLHLS), an extensive ongoing investigation primarily focused on older adults across various provinces in China. The CLHLS was initiated in 1998 and follow-up surveys were conducted in 2000, 2002, 2005, 2008, 2011, 2014, and 2018. At its inception, the survey covered half of the counties or municipalities in 22 out of China’s 31 provinces. In the 2014 wave, it expanded its coverage to all 23 provinces, including Chengmai City in Hainan Province. The dataset includes individual-level information on demographics, health indicators, socioeconomic characteristics, and social and behavioral risk factors. It upholds high-quality data integrity, as confirmed by systematic evaluations ensuring randomness in attrition, credibility in measurement scales, and accuracy in reporting ages. A comprehensive description of CLHLS can be found elsewhere [11, 12].

For this study, baseline data were extracted from the 2014 wave of CLHLS because only the 2018 wave collected data on cause-specific deaths. The 2014 wave encompassed 7,192 individuals, achieving a follow-up response rate of 79.0% during the 2018 wave. To maintain focus on older adults, exclusion criteria were applied, which excluded participants younger than 65 years old (*n* = 85), those lost to follow-up (*n* = 1,511), individuals with missing psychological resilience data (*n* = 658), and cases with incorrect death dates (*n* = 3). Ultimately, 4,935 participants were included for analysis. Detailed information on the inclusion and exclusion criteria is provided in Supplementary Fig. 1. This study was approved by the Biomedical Ethics Committee of Peking University (IRB00001052-13074).

### Assessment of psychological resilience

Psychological resilience was assessed using a five-item scale adapted from previous research [13–15]. The scale was designed to capture various dimensions of resilience pertinent to older adults, including optimism, coping strategies, emotional well-being, and decision-making autonomy. The specific items included in the scale were: “Feel the older you get, the more useless you are”, “Look on the bright side of things”, “Feel fearful or anxious”, “Feel lonely and isolated”, and “Make own decisions concerning personal affairs.” Participants rated their responses on a five-point scale ranging from “Always” to “Never”. Individual item scores were summed to generate a total resilience score, ranging from 5 to 25, with higher scores indicating greater psychological resilience (Supplementary Table 1). These items have been validated in previous studies involving older populations, demonstrating their relevance and sensitivity in measuring psychological resilience [13–15]. To categorize participants, psychological resilience was dichotomized into low (below the median) and high (above the median) levels based on the median score. Dichotomizing the psychological resilience score simplifies statistical analysis and facilitates comparisons between groups with differing resilience levels. Clinically, it aids in identifying individuals with low resilience for targeted interventions. This method aligns with previous research practice, enhancing comparability across study [13]. Using the median score as the cut-off ensures balanced group sizes and does not assume a specific score distribution, thereby improving statistical power in comparative analyses.

### Outcome assessment

The classification of causes of mortality was conducted following the International Statistical Classification of Diseases, 10th revision (ICD-10). The primary outcomes of interest were mortality from all causes and specific causes, including cardiovascular diseases (CVD) (ICD-10 codes I00-I99), respiratory diseases (ICD-10 codes J00–99), cancer (ICD-10 codes C00-C97), and other causes. Information regarding reported deaths was obtained from the next of kin of study participants. Survival time was calculated from the date of the first interview until either the date of death or the last follow-up assessment.

### Covariates assessment

We incorporated a total of 23 covariates to comprehensively account for baseline sociodemographic characteristics, lifestyle behaviors, and health status to control for potential confounders. The covariates were assessed only at baseline. The sociodemographic segment encompassed various facets, including age, gender (male or female), education level (0 years, 1–6 years, or > 6 years), residential setting (rural area or urban area), marital status (married or other statuses - divorced, widowed, or never married), living arrangements (living with family, living alone, or living in institution), and economic status (dependent or independent). Lifestyle behaviors were examined across multiple dimensions, encompassing smoking habits (never, current, or former), alcohol consumption (never, current, or former), regularity of exercise (never, current, or former), as well as the frequency of vegetable intake (daily, quite often, occasionally, rarely, or none), fruit intake (daily, quite often, occasionally, rarely, or none), meat consumption (daily, weekly, monthly, occasionally, rarely, or none), fish consumption (daily, weekly, monthly, occasionally, rarely, or none), and egg consumption (daily, weekly, monthly, occasionally, rarely, or none). The health characteristics section comprised body mass index (BMI), sleep time (< 6 h, 6 to 9 h, or ≥ 9 h), self-reported doctor-diagnosed conditions, including hypertension (yes or no), heart disease (yes or no), cerebrovascular disease (yes or no), diabetes mellitus (yes or no), respiratory diseases (encompassing pneumonia, bronchitis, emphysema, and asthma) (yes or no), and cancer (yes or no). BMI was categorized as underweight (< 18.5 kg/m^2^), normal (18.5–23.9 kg/m^2^), overweight (24–27.9 kg/m^2^), or obese (≥ 28 kg/m^2^) [11]. The selection of covariates was guided by their established or potential influence on the outcomes under study, reflecting both theoretical considerations and empirical evidence from prior research [16–24]. The rationale for including these nutritional variables is grounded in their significant role in influencing overall health status, disease prevention, and longevity [25, 26].

### Statistical analysis

Baseline characteristics of the study population were summarized as means and standard deviations (SDs) for continuous variables and as percentages for categorical variables. Missing data were addressed using Multiple Imputation by Chained Equations with predicted mean matching to ensure robust handling of the data gaps. The decision to use five imputations was based on Rubin’s rule, which suggests that a small number of imputations (typically between 3 and 10) is often sufficient to achieve stable and reliable estimates [27]. This approach enhances the robustness of our analysis by reducing the bias and increasing the efficiency of our statistical estimates. Detailed information on the missing variables is provided in Supplementary Table 2. The use of five imputations was chosen to balance computational efficiency with the need for accurate imputation, as it has been shown that increasing the number of imputations beyond five often yields diminishing returns in terms of statistical power and precision [28].

We tested the proportional hazards assumption using the Schoenfeld residual test, finding no evidence of violation of this assumption. Kaplan-Meier survival analysis was utilized to construct survival curves for psychological resilience, with differences between groups assessed via log-rank testing. We calculated tolerance values and variance inflation factors (VIF) for each covariate. Our criteria for acceptable multicollinearity were tolerance values greater than 0.1 and VIF values less than 10. All covariates met these criteria, indicating the absence of significant multicollinearity. Cox proportional hazard models were applied to examine the association of psychological resilience with all-cause and cause-specific mortality, reported as hazard ratios (HRs) with corresponding 95% confidence intervals (CIs). Model 1 was adjusted for baseline age and sex. Model 2 was further adjusted for a comprehensive set of potential confounders, including marital status, education level, residence, living arrangement, economic status, smoking status, alcohol consumption, regular exercise, sleep time, BMI, and prevalent medical conditions (hypertension, heart disease, cerebrovascular disease, diabetes mellitus, respiratory disease, and cancer). Model 3 extended the adjustment variables to encompass dietary factors such as intake frequency of fruits, vegetables, meat, fish, and eggs, in addition to the variables in Model 2. The crude incidence rate (IR) of all-cause and cause-specific mortality was estimated per 1000 person-years. Additionally, a restricted cubic spline analysis was conducted to assess the dose-response relationship between changes in psychological resilience scores and the risk of mortality. This approach offers several advantages over linear or categorical analyses. Firstly, it allows for the assessment of potential non-linear relationships between variables, which may better capture complex associations present in the data. Secondly, it provides flexibility in modeling the shape of the dose-response curve, thus enhancing the accuracy of risk estimation. We set the reference values at the 50th percentile for psychological resilience scores (19 scores), using four knots at specific percentiles of their distributions (5th, 35th, 65th, and 95th). These knots were selected to ensure sufficient flexibility in capturing potential non-linear relationships while maintaining statistical robustness [29]. Moreover, we performed interaction and subgroup analyses by sex, age, marital status, residence, living arrangement, economic status, and BMI within a multivariable-adjusted model.

To ensure robustness, sensitivity analyses were conducted. Firstly, complete case analyses were performed, excluding participants with incomplete covariate data. Secondly, individuals with prevalent major chronic diseases (including heart disease, diabetes mellitus, cerebrovascular disease, respiratory disease, or cancer) at baseline were excluded to explore potential reverse causality. Thirdly, to mitigate the potential impact of short-term follow-up, we excluded deaths occurring within the first year. This decision was made considering that the effects of psychological resilience on mortality typically unfold over a chronic timeframe. Finally, we employed propensity score matching (PSM) to ensure a balanced distribution of baseline characteristics among groups. Matching on a 1:1 ratio based on the propensity score was carried out using a nearest neighbor-matching algorithm, with a maximum caliper set at 0.05 of the propensity score. Adequate balance between groups was considered achieved when the standardized mean difference (SMD) was less than 0.1 [30].

All statistical analyses were executed using R statistical software version 4.2.2 (R Foundation for Statistical Computing) with a significance threshold set at a two-tailed *P* value < 0.05.

## Results

### Characteristics of study participants

A total of 4935 older adults (52.0% female) with a mean age of 84.27 (10.02) years were included. Table 1 outlines the demographic and lifestyle characteristics of the study population. Participants reporting high levels of psychological resilience (51.7%) were typically younger, more often male, urban residents, married, living with family, literate, economically independent, smokers, alcohol consumers, engaged in regular exercise, possessed a higher BMI, experienced longer sleep duration, and reported sufficient intake of vegetables, fruits, meat, fish, and eggs, compared to those with lower levels of psychological resilience. Moreover, this group showed a lower incidence of cerebrovascular diseases.

**Table 1:** Baseline characteristics of the participants

Characteristics	Total (*n* = 4935)	Psychological resilience		*P* value
		Low level (*n* = 2384)	High level (*n* = 2551)	
Age (year), mean (SD)	84.27 (10.02)	85.92 (10.07)	82.72 (9.73)	< 0.001
Female, no. (%)	2564 (52.0)	1390 (58.3)	1174 (46.0)	< 0.001
Urban area, no. (%)	2137 (43.3)	903 (37.9)	1234 (48.4)	< 0.001
Married, no. (%)	2141 (43.4)	823 (34.5)	1318 (51.7)	< 0.001
living arrangement, no. (%)				< 0.001
Living with family	3838 (77.8)	1799 (75.5)	2039 (79.9)	
Living alone	1002 (20.3)	529 (22.2)	473 (18.5)	
Living in institution	95 (1.9)	56 (2.3)	39 (1.5)	
Education (year), no. (%)				< 0.001
0	2702 (54.8)	1523 (63.9)	1179 (46.2)	
1–6	1681 (34.1)	693 (29.1)	988 (38.7)	
> 6	552 (11.2)	168 (7.0)	384 (15.1)	
Economic independence, no. (%)	1319 (26.7)	437 (18.3)	882 (34.6)	< 0.001
Smoking status, no. (%)				< 0.001
Never	3433 (69.6)	1799 (75.5)	1634 (64.1)	
Current	848 (17.2)	349 (14.6)	499 (19.6)	
Former	654 (13.3)	236 (9.9)	418 (16.4)	
Drinking status, no. (%)				< 0.001
Never	3650 (74.0)	1887 (79.2)	1763 (69.1)	
Current	786 (15.9)	286 (12.0)	500 (19.6)	
Former	499 (10.1)	211 (8.9)	288 (11.3)	
Regular exercise, no. (%)				< 0.001
Never	3344 (67.8)	1861 (78.1)	1483 (58.1)	
Current	1372 (27.8)	413 (17.3)	959 (37.6)	
Former	219 (4.4)	110 (4.6)	109 (4.3)	
BMI (kg/m^2^), no. (%)				
Underweight (< 18.5)	2729 (55.3)	1270 (53.3)	1459 (57.2)	
Normal (18.5–24)	899 (18.2)	556 (23.3)	343 (13.4)	
Overweight (24–28)	991 (20.1)	424 (17.8)	567 (22.2)	
Obese (≥ 28)	316 (6.4)	134 (5.6)	182 (7.1)	
Sleep time (h), no. (%)				< 0.001
< 6	814 (16.5)	445 (18.7)	369 (14.5)	
6–9	2803 (56.8)	1339 (56.2)	1464 (57.4)	
≥ 9	1318 (26.7)	600 (25.2)	718 (28.1)	
Intake of fruit, no. (%)				< 0.001
Daily	657 (13.3)	233 (9.8)	424 (16.6)	
Quite often	1390 (28.2)	631 (26.5)	759 (29.8)	
Occasionally	1701 (34.5)	902 (37.8)	799 (31.3)	
Rarely or none	1187 (24.1)	618 (25.9)	569 (22.3)	
Intake of vegetables, no. (%)				< 0.001
Daily	2820 (57.1)	1206 (50.6)	1614 (63.3)	
Quite often	1592 (32.3)	855 (35.9)	737 (28.9)	
Occasionally	389 (7.9)	249 (10.4)	140 (5.5)	
Rarely or none	134 (2.7)	74 (3.1)	60 (2.4)	
Intake of meat, no. (%)				< 0.001
Daily	1897 (38.4)	829 (34.8)	1068 (41.9)	
Weekly	2079 (42.1)	1032 (43.3)	1047 (41.0)	
Monthly	384 (7.8)	224 (9.4)	160 (6.3)	
Occasionally	258 (5.2)	144 (6.0)	114 (4.5)	
Rarely or none	317 (6.4)	155 (6.5)	162 (6.4)	
Intake of fish, no. (%)				< 0.001
Daily	380 (7.7)	159 (6.7)	221 (8.7)	
Weekly	2075 (42.0)	961 (40.3)	1114 (43.7)	
Monthly	1020 (20.7)	550 (23.1)	470 (18.4)	
Occasionally	643 (13.0)	319 (13.4)	324 (12.7)	
Rarely or none	817 (16.6)	395 (16.6)	422 (16.5)	
Intake of egg, no. (%)				
Daily	1428 (28.9)	535 (22.4)	893 (35.0)	< 0.001
Weekly	1996 (40.4)	1022 (42.9)	974 (38.2)	
Monthly	695 (14.1)	402 (16.9)	293 (11.5)	
Occasionally	358 (7.3)	200 (8.4)	158 (6.2)	
Rarely or none	458 (9.3)	225 (9.4)	233 (9.1)	
Hypertension, no. (%)	1720 (34.9)	827 (34.7)	893 (35.0)	0.84
Heart disease, no. (%)	675 (13.7)	346 (14.5)	329 (12.9)	0.11
Diabetes mellitus, no. (%)	292 (5.9)	126 (5.3)	166 (6.5)	0.08
Cerebrovascular disease, no. (%)	427 (8.7)	243 (10.2)	184 (7.2)	< 0.001
Respiratory disease, no. (%)	601 (12.2)	294 (12.3)	307 (12.0)	0.78
Cancer, no. (%)	43 (0.9)	25 (1.0)	18 (0.7)	0.25

*BMI* Body Mass Index

Notes: Values were presented as number (%) or mean ± SD. Differences in characteristics were compared using the χ^2^ test for categorical variables and t-test for continuous variables

After implementing PSM, our analysis retained 3368 participants. The SMDs of the covariates were consistently below 0.1, as illustrated in Supplementary Fig. 2. Furthermore, examination of baseline variables in Supplementary Table 3 revealed a high degree of balance between the two groups.

### Association of psychological resilience with all-cause and cause-specific mortality

Over a median follow-up of 3.5 years (interquartile range: 2.7 to 4.2, totaling 16,016.75 person-years), a total of 1,726 participants (35.0%) experienced mortality. Among these deaths, 354 (20.5%) were attributed to CVD, 190 (11.0%) to respiratory diseases, 104 (6.0%) to cancer, and 1,078 (62.5%) to other causes.

**Fig. 1:**
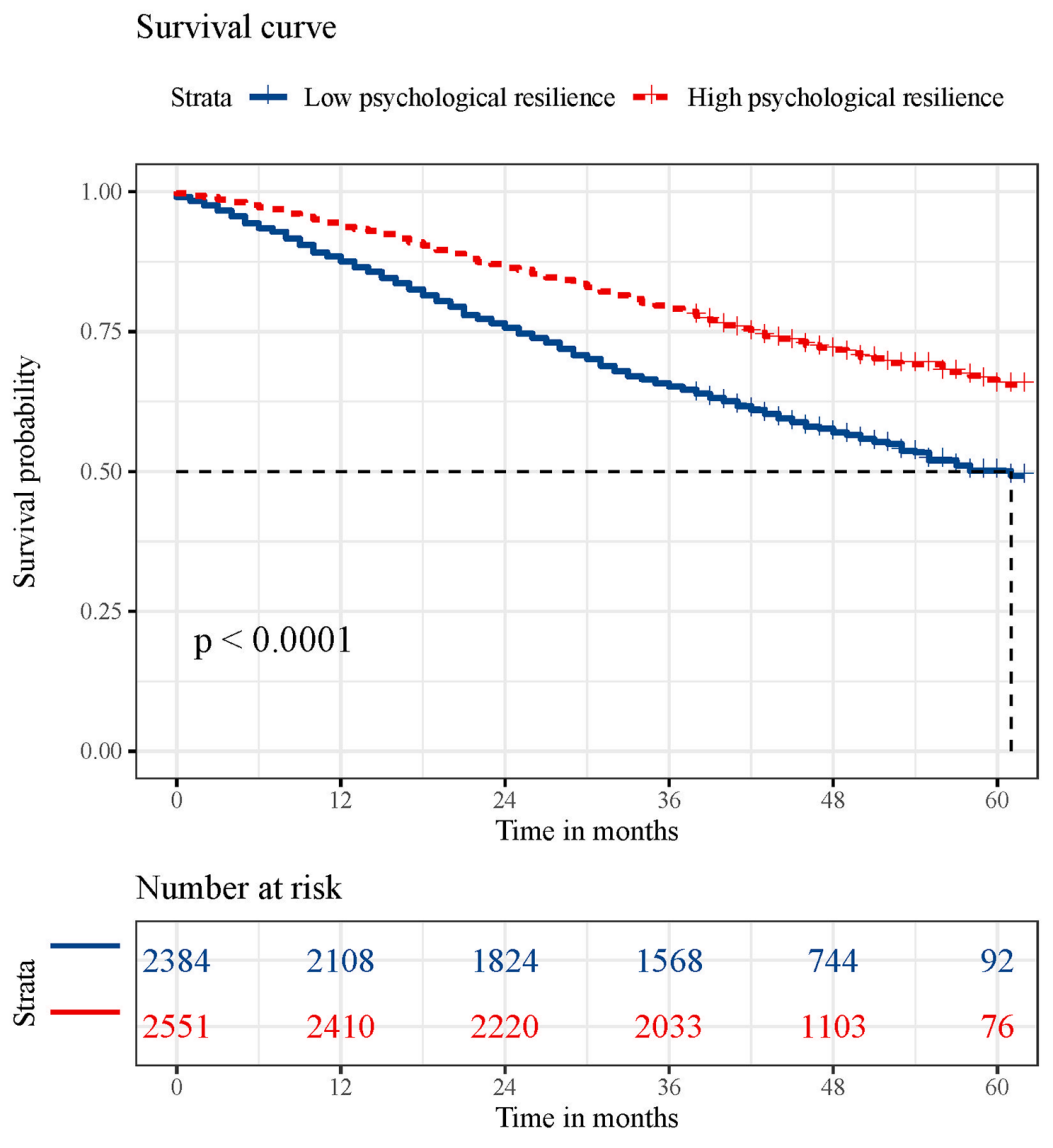
Kaplan-Meier survival curves for all-cause mortality according to psychological resilience. The median survival duration is represented using a vertical dashed line

Kaplan-Meier curves revealed significant survival differences linked to psychological resilience levels (log-rank test: *p* < .001) (Fig. 1). Specifically, those with low psychological resilience showed markedly lower survival rates. In comparison to those with low psychological resilience, individuals exhibiting high psychological resilience demonstrated a substantially reduced risk of all-cause mortality (HR 0.74, 95% CI 0.67–0.82) (Table 2). Cause-specific analyses revealed significant associations between high psychological resilience and reduced mortality from CVD (HR 0.74, 95% CI 0.59–0.93), respiratory diseases (HR 0.63, 95% CI 0.45–0.87), and other causes (HR 0.69, 95% CI 0.60–0.78), but not from cancer (Table 2).

**Table 2:** Hazard ratios for all-cause and cause-specific mortality according to psychological resilience

Characteristic	Number Of deaths (Incidence rate ^a^)	Unadjusted Model	Model 1	Model 2	Model 3
		HR (95% CI)	HR (95% CI)	HR (95% CI)	HR (95% CI)
All-cause mortality					
Low psychological resilience	1010 (139.6)	Reference	Reference	Reference	Reference
High psychological resilience	716 (81.5)	0.58 (0.53–0.64)	0.66 (0.60–0.73)	0.74 (0.67–0.82)	0.74 (0.67–0.82)
Psychological resilience score		0.90 (0.88–0.91)	0.92 (0.90–0.93)	0.94 (0.92–0.95)	0.94 (0.92–0.95)
Cardiovascular disease mortality					
Low psychological resilience	205 (35.8)	Reference	Reference	Reference	Reference
High psychological resilience	149 (19.7)	0.55 (0.45–0.68)	0.62 (0.50–0.77)	0.76 (0.60–0.95)	0.74 (0.59–0.93)
Psychological resilience score		0.89 (0.86–0.92)	0.91 (0.88–0.94)	0.94 (0.91–0.98)	0.94 (0.90–0.97)
Respiratory disease mortality					
Low psychological resilience	115 (20.6)	Reference	Reference	Reference	Reference
High psychological resilience	75 (10.1)	0.49 (0.37–0.66)	0.52 (0.39–0.70)	0.59 (0.43–0.81)	0.63 (0.45–0.87)
Psychological resilience score		0.87 (0.83–0.91)	0.88 (0.84–0.92)	0.90 (0.86–0.95)	0.91 (0.86–0.96)
Cancer mortality					
Low psychological resilience		Reference	Reference	Reference	Reference
High psychological resilience	38 (7.0)	1.27 (0.85–1.89)	1.19 (0.79–1.78)	1.17 (0.76–1.81)	1.07 (0.69–1.67)
Psychological resilience score	66 (8.9)	1.01 (0.95–1.08)	0.99 (0.93–1.06)	0.98 (0.91–1.05)	0.96 (0.90–1.03)
Other cause mortality					
Low psychological resilience	652 (99.0)	Reference	Reference	Reference	Reference
High psychological resilience	426 (52.0)	0.52 (0.46–0.59)	0.61 (0.54–0.69)	0.69 (0.61–0.78)	0.69 (0.60–0.78)
Psychological resilience score		0.88 (0.86–0.89)	0.90 (0.89–0.92)	0.92 (0.90–0.94)	0.92 (0.90–0.94)

*HR* hazard ratio; *CI* confidence interval

Notes: ^a^ Incidence rates per 1000 person-years

Model 1: adjusted for baseline age and sex

Model 2: further adjusted for marital status, education, residence, living arrangement, economic status, smoking status, drinking status, regular exercise, sleep time, body mass index, hypertension, heart disease, cerebrovascular disease, diabetes mellitus, respiratory disease, and cancer

Model 3: further adjusted for intake of fruit, intake of vegetables, intake of meat, intake of fish, and intake of egg

For each incremental unit increase in psychological resilience score, there was a corresponding reduction in the HRs for various causes of mortality. Specifically, higher psychological resilience scores were associated with decreased risks for all-cause mortality (HR 0.94, 95% CI 0.92–0.95), CVD mortality (HR 0.94, 95% CI 0.90–0.97), respiratory disease mortality (HR 0.91, 95% CI 0.86–0.96), and mortality from other causes (HR 0.92, 95% CI 0.90–0.94) (Table 2).

**Fig. 2:**
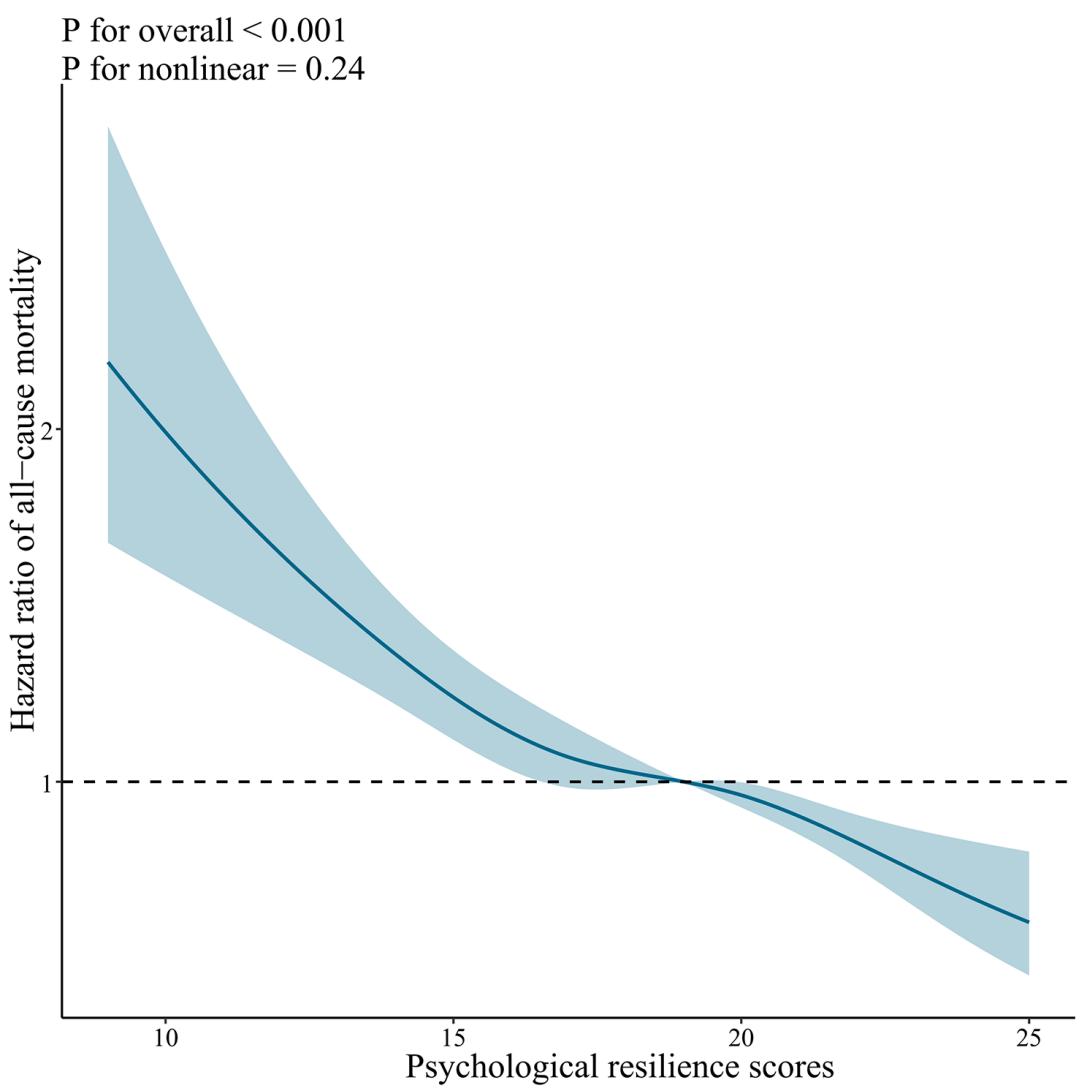
Dose-response association between psychological resilience and risk of all-cause mortality. Notes: Solid red lines are multivariable-adjusted hazard ratios, with shaded areas showing 95% confidence intervals derived from restricted cubic spline regressions with four knots. Multivariate models were adjusted for baseline age, sex, marital status, education, residence, living arrangement, economic status, smoking status, drinking status, regular exercise, sleep time, body mass index, hypertension, heart disease, cerebrovascular disease, diabetes mellitus, respiratory disease, cancer, intake of fruit, intake of vegetable, intake of meat, intake of fish, and intake of egg

Furthermore, by employing the restricted cubic spline method, we identified a progressive reduction in the risks of all-cause mortality, CVD mortality, respiratory disease mortality, and mortality from other causes as scores in psychological resilience increased (Fig. 2 and Supplemental Fig. 3).

### Subgroup analyses

**Fig. 3:**
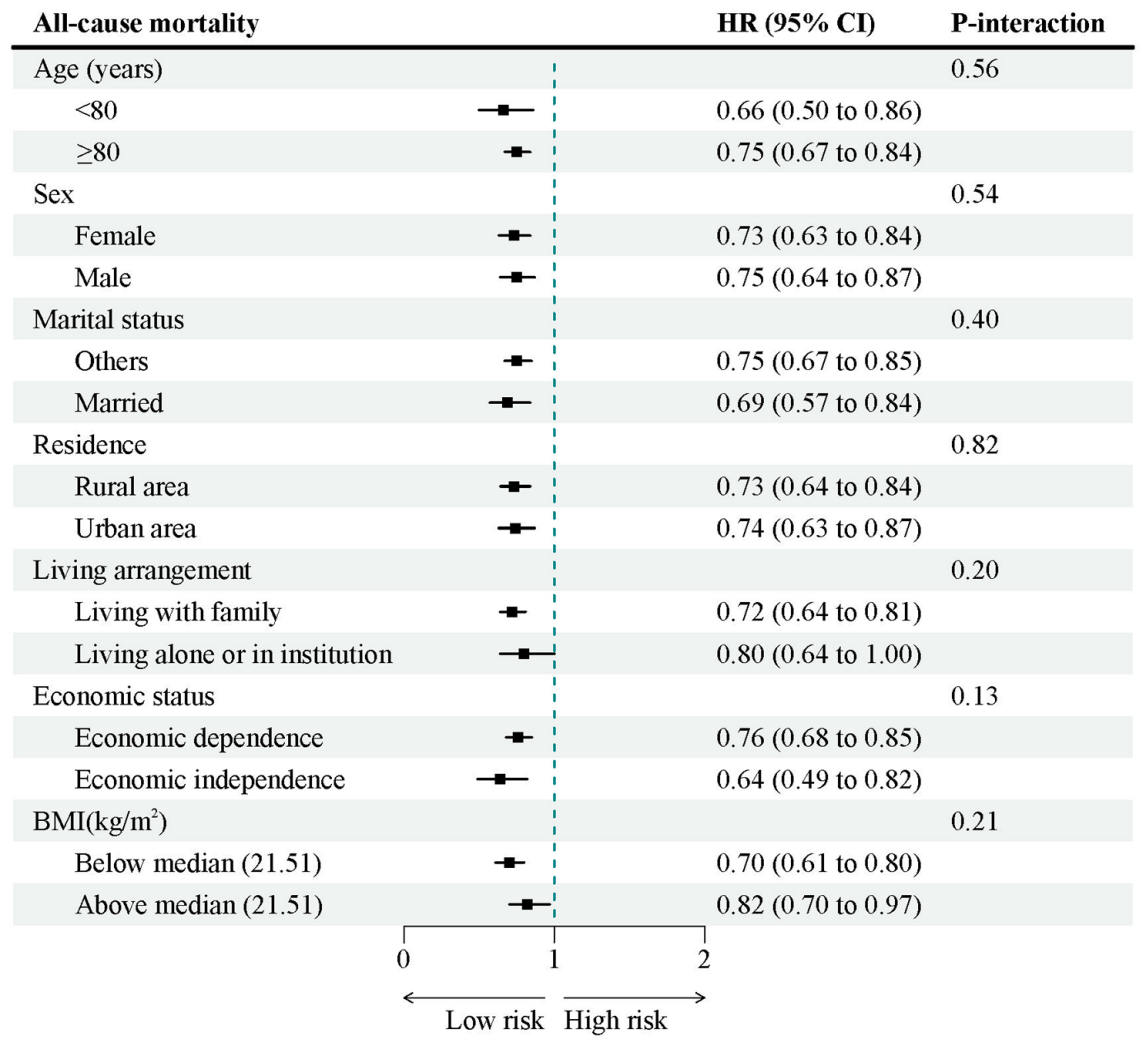
Association of psychological resilience with all-cause mortality stratified by participant characteristics. *HR* hazard ratio; *CI* confidence interval. Notes: Each stratification controlled for all factors (baseline age, sex, marital status, education, residence, living arrangement, economic status, smoking status, drinking status, regular exercise, sleep time, body mass index, hypertension, heart disease, cerebrovascular disease, diabetes mellitus, respiratory disease, cancer, intake of fruit, intake of vegetable, intake of meat, intake of fish, and intake of egg) except the stratification factor itself

We conducted stratified analyses to examine the relationships between psychological resilience and all-cause as well as cause-specific mortality concerning potential risk factors, utilizing a fully adjusted model (Fig. 3 and Supplemental Fig. 4). We observed a notably stronger impact of psychological resilience on other-cause mortality among economically independent older adults compared to their economically dependent counterparts (*P*-value for interaction = 0.02). However, none of the other variables examined, including age, sex, marital status, residence, living arrangement, and BMI, significantly modified the relationships between psychological resilience and different causes of mortality.

### Sensitivity analyses

To validate our findings, we conducted sensitivity analyses (Supplementary Table 4). Even after excluding participants with missing covariate values, the relationships between psychological resilience and both all-cause and cause-specific mortality remained consistent. Furthermore, excluding participants with pre-existing conditions such as heart disease, diabetes mellitus, cerebrovascular disease, respiratory disease, and cancer did not significantly alter the study’s conclusions. Similarly, excluding participants who died within one year of follow-up did not produce noteworthy changes in the outcomes. Lastly, the results obtained from PSM were consistent with those from our primary analysis.

## Discussion

The findings of this cohort study provide significant insights into the association of psychological resilience with all-cause and cause-specific mortality in older adults. The independent associations between higher psychological resilience and decreased risks of all-cause mortality, CVD mortality, respiratory disease mortality, and mortality from other causes, excluding cancer-related mortality, underscore the potential importance of psychological factors in promoting healthy aging.

Previous studies in China have investigated the relationship between psychological resilience and longevity among centenarians and elderly individuals over 80 years old, revealing a negative correlation between high psychological resilience and all-cause mortality [4, 5]. These studies highlighted the potential role of psychological factors in promoting longevity but primarily focused on this specific age group. In our study, we extended the investigation to elderly individuals aged over 65 years. We found that psychological resilience was negatively associated with all-cause mortality. Subgroup analysis based on age revealed that this negative association was held for both those aged 80 years and above and those aged below 80 years. These findings replicate and expand upon previous research, underscoring the importance of psychological resilience in promoting longevity across a broader age range. In addition, our study goes beyond examining cause-specific mortality, revealing independent associations with lower mortality from CVD, respiratory diseases, and other causes. The observed distinctions in cause-specific mortality further contribute to this nuanced understanding, revealing varying associations with different causes of death. Nevertheless, there was a lack of a clear association between psychological resilience and cancer-related mortality. This discovery contrasts with an earlier study that implied possible connections between resilience and cancer outcomes [31], necessitating further investigation into potential moderating factors or mechanisms influencing these findings. Subsequent research could explore whether certain psychosocial variables or specific types of cancer within the broader category of cancer mortality show varied associations with psychological resilience.

In addition, we investigated the intriguing phenomenon of a notably stronger impact of psychological resilience on other-cause mortality among economically independent older adults in comparison to their economically dependent counterparts. Our findings clarify that economic status moderated the association of psychological resilience specifically with other-cause mortality, not with mortality in general. This distinction is crucial as it highlights that the protective effects of psychological resilience are more pronounced in non-specific causes of death among those who are financially independent. This phenomenon can be attributed to the increased autonomy and decision-making capabilities that come with financial self-sufficiency. Economic independence fosters a sense of control and purpose, thereby reinforcing psychological well-being [32–34] as a protective factor against mortality. Additionally, self-reliant individuals are more likely to adopt health-promoting behaviors and employ adaptive coping strategies [35, 36], which further enhances their resilience. In contrast, older adults who are economically dependent may face additional stressors related to financial instability [37], potentially reducing the impact of psychological resilience on mortality. Understanding these dynamics is crucial for developing targeted interventions aimed at bolstering resilience among vulnerable populations. By focusing on economically dependent individuals, who may not experience the full protective effects of psychological resilience, we can devise strategies to enhance their psychological well-being and ultimately promote healthy aging and longevity.

The potential mechanisms through which psychological resilience influences mortality are diverse and multifaceted. Our results show a significant association between higher psychological resilience and reduced CVD mortality. This finding aligns with previous research suggesting that psychological resilience can mitigate the adverse effects of stress and inflammation, which are critical factors in the development and progression of CVD [10]. Resilient individuals often have better stress management skills, leading to lower levels of stress hormones such as cortisol [38], which in turn reduces the risk of hypertension, atherosclerosis, and other cardiovascular conditions [39, 40]. Additionally, resilient individuals are more likely to engage in heart-healthy behaviors, such as regular exercise, a balanced diet, and adherence to medical advice [7]. These behaviors play a crucial role in preventing CVD and improving outcomes for those already diagnosed with the disease [41]. The association between higher psychological resilience and lower respiratory disease mortality highlights another critical pathway through which resilience can influence health outcomes. Chronic respiratory conditions, such as chronic obstructive pulmonary disease and asthma, are exacerbated by stress and poor mental health [42]. Resilient individuals typically experience lower levels of stress and are better equipped to cope with chronic illnesses, which can lead to better disease management and improved survival rates. Psychological resilience may influence respiratory health through immunomodulation, as resilience has been linked to enhanced immune function, including the production of anti-inflammatory cytokines [10, 43]. This immunomodulatory effect may contribute to a reduced risk of respiratory infections and subsequently lower mortality from respiratory diseases. Moreover, psychological resilience has been linked to better adherence to treatment regimens and preventive measures, such as vaccinations [44] and avoiding smoking [45, 46], which are essential for managing respiratory diseases and preventing severe complications. Our study also found a significant association between higher psychological resilience and reduced mortality from other causes. This broad category encompasses various health conditions, many of which can be influenced by the psychosocial and behavioral factors associated with resilience. For instance, resilient individuals often have stronger social support networks [47], which can provide emotional and practical assistance during health crises, leading to better health outcomes and survival rates. Interestingly, our findings indicate that psychological resilience is associated with lower all-cause and cause-specific mortality, except for cancer-related mortality. This discrepancy might be explained by the unique nature of cancer’s etiology and progression. Unlike cardiovascular diseases or external causes where psychological factors might directly influence lifestyle choices and stress responses, cancer progression could be more heavily influenced by genetic factors, biological processes, and environmental exposures that resilience does not significantly alter. Moreover, the impact of resilience on health behaviors, while beneficial for general well-being, might not be sufficient to counteract the complex pathophysiological mechanisms specific to cancer development and progression. Further research is needed to explore these differential impacts and understand the underlying biological and psychological interactions.

### Strengths and limitations

The strengths of this study lie in its longitudinal design, large sample size, and comprehensive assessment of various causes of mortality, enabling a nuanced understanding of the relationship between psychological resilience and mortality risks among older adults. However, several limitations should be acknowledged. First, the study’s reliance on self-reported measures of psychological resilience introduces potential recall bias and subjective interpretation. Future research could benefit from objective measures or longitudinal assessments to provide a more nuanced understanding of these constructs and their impact on mortality. Second, the study’s observational nature precludes establishing causality, warranting caution in inferring direct causal relationships between psychological resilience and mortality outcomes. Longitudinal interventional studies exploring the effects of targeted interventions aimed at enhancing psychological resilience among older adults could provide further insights into the causal pathways linking these factors to mortality outcomes. Third, residual confounding factors, such as underlying health conditions or unmeasured socioeconomic variables, could have influenced the observed associations. Fourth, the cohort’s homogeneity in terms of demographic characteristics and geographic location might limit the generalizability of our findings to more diverse populations. Finally, while our study treated psychological resilience as a time-invariant variable, its dynamic nature warrants acknowledgment. Future research should explore the evolving aspect of resilience over time to better understand its association with mortality in older adults. Integrating longitudinal designs or dynamic measures of psychological resilience could offer deeper insights into its impact on health outcomes.

## Conclusion

Our cohort study provides robust evidence supporting the independent associations between higher psychological resilience and decreased risks of all-cause, CVD, respiratory disease, and other cause mortality in older adults. These findings underscore the potential importance of psychological factors in promoting healthy aging and advocate for the integration of psychological resilience assessments in comprehensive geriatric care. Future research should delve into the mechanisms underlying these associations, paving the way for targeted interventions to enhance psychological resilience and, consequently, improve overall mortality outcomes in the aging population.

### Electronic supplementary material

Below is the link to the electronic supplementary material.

Supplementary Material 1

## Data Availability

The dataset supporting the conclusions of this article is available in the (Peking University Open Research Data Platform) repository, (unique persistent identifier and hyperlink to dataset in https://opendata.pku.edu.cn/dataset.xhtml? persistentId=doi:10.18170/DVN/WBO7LK).

## Abbreviations

CLHLS: Chinese Longitudinal Healthy Longevity Survey
BMI: Body mass index
HR: Hazard ratio
CI: Confidence interval
ICD-10: International Statistical Classification of Diseases,10th revision
CVD: Cardiovascular disease
SD: Standard deviation
PSM: Propensity score matching
SMD: Standardized mean difference
