# Supplementary material for: Association of psychological resilience with all-cause and cause-specific mortality in older adults: a cohort study
Source: BMC Public Health. 2024 Jul 25;24:1989. doi: 10.1186/s12889-024-19558-8 (PMC11270806; doi:10.1186/s12889-024-19558-8)
Supplement: Supplementary file 1 — Supplementary Material 1 [file 12889_2024_19558_MOESM1_ESM.docx]

**Supplementary Material**

**Supplementary Figure 1** Flowchart of the included study population.

**Supplementary Figure 2** The standardized mean differences (SMD) of the variables.

**Supplemental Figure 3** Dose-response association between psychological resilience and cause-specific mortality.

**Supplemental Figure 4** Association of psychological resilience with cause-specific mortality stratified by participant characteristics.

**Supplementary Table 1** The coding of psychological resilience.

**Supplementary Table 2** The numbers (percentage) of the missing variables.

**Supplementary Table 3** Demographic and clinical characteristics of the study population after propensity score matching.

**Supplementary Table 4** Sensitivity analyses for the association of psychological resilience with all-cause and cause-specific mortality risk


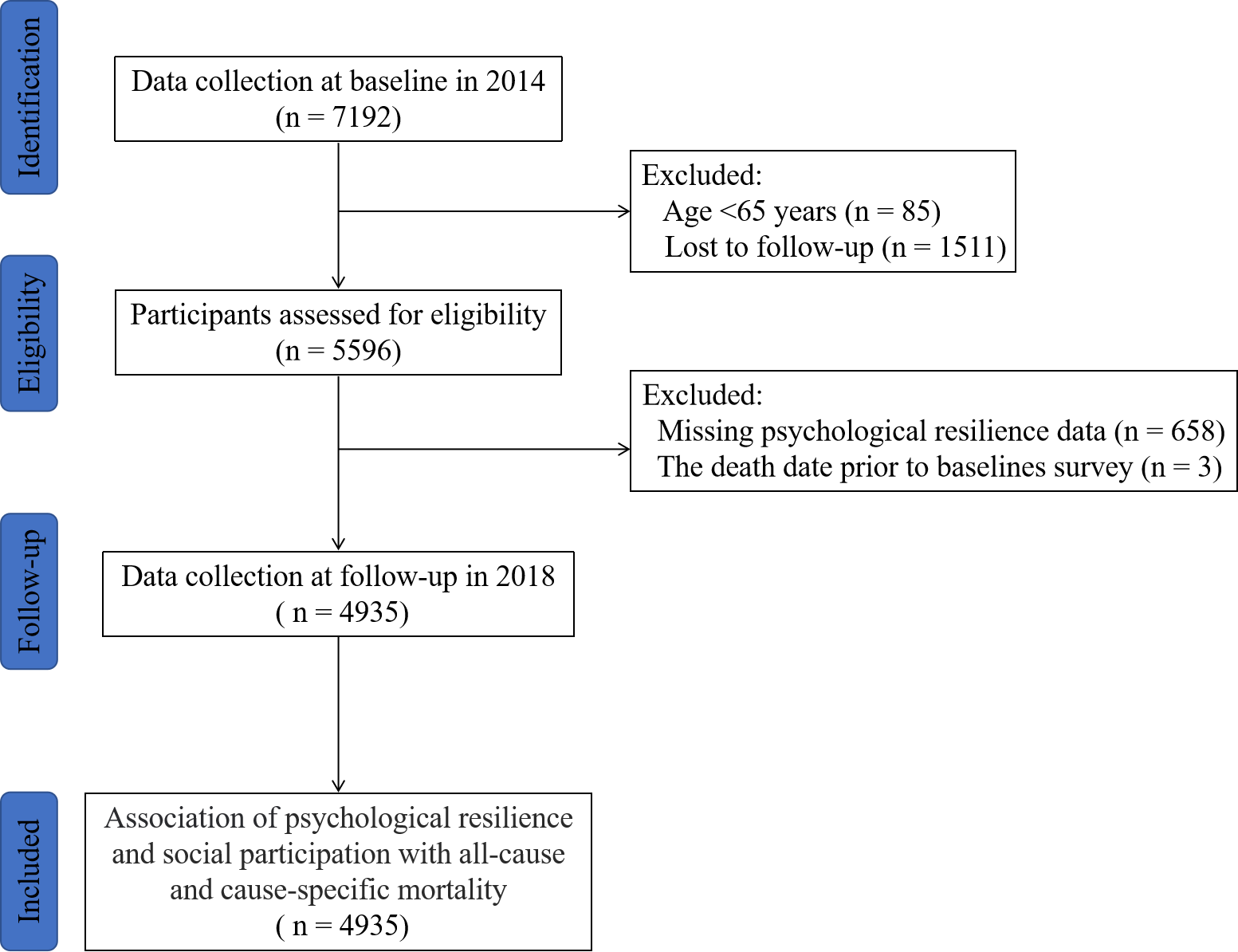


**Supplemental Figure 1** Flowchart of the included study population.


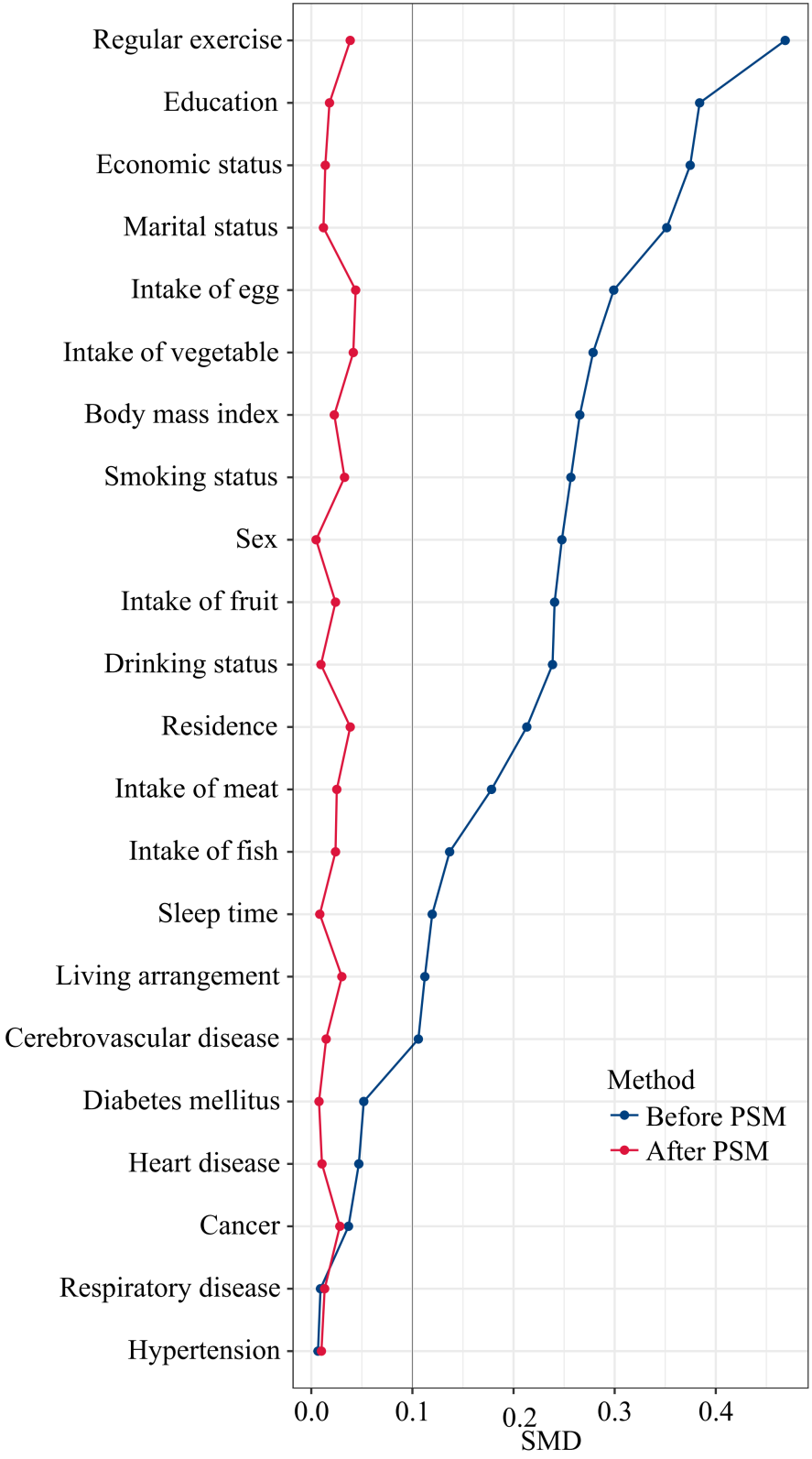


**Supplementary Figure 2** The standardized mean differences (SMD) of the variables.

Notes: *PSM* propensity score matching.


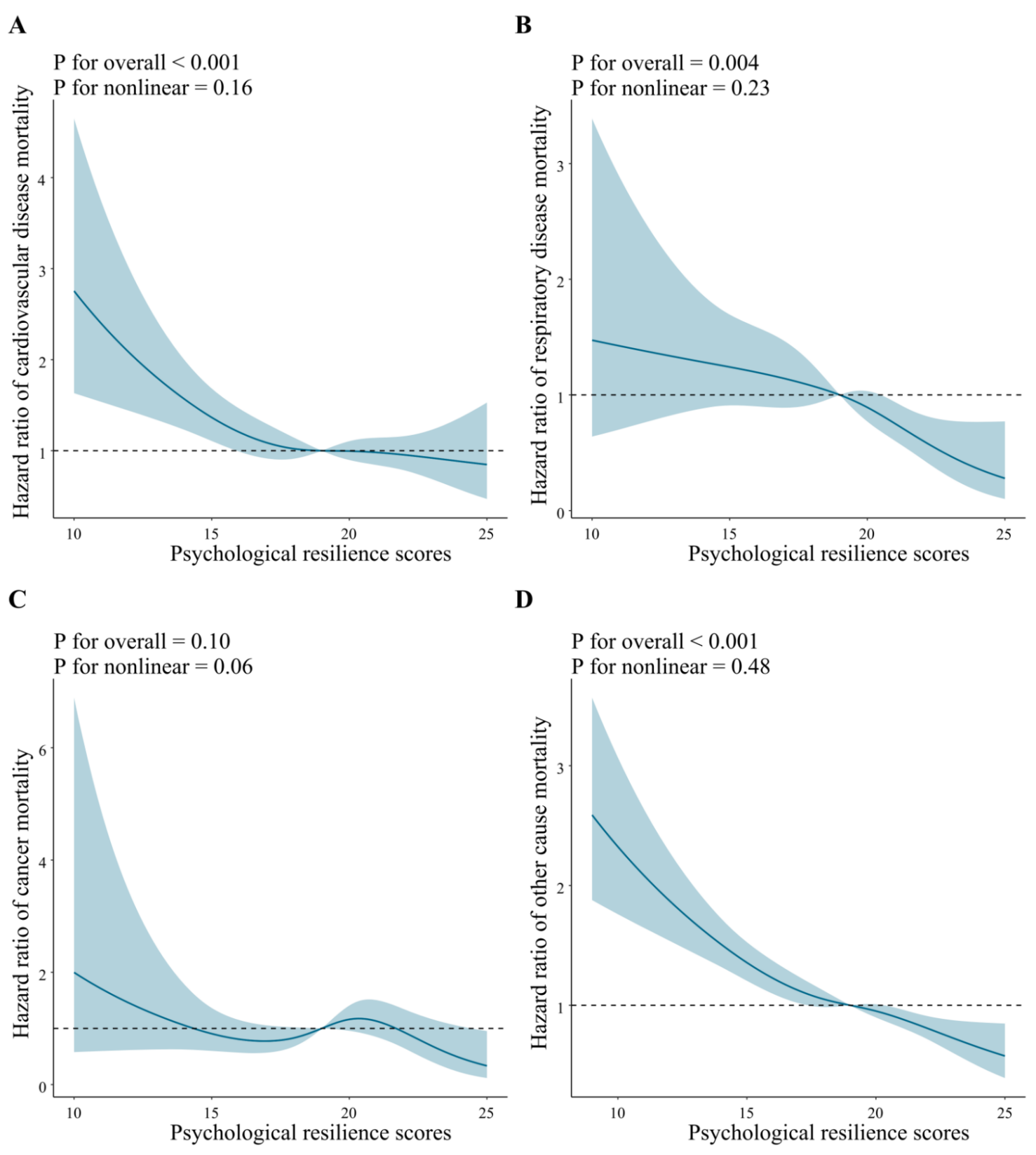


**Supplemental Figure 3** Dose-response association between psychological resilience and cause-specific mortality.

Notes: Solid blue lines are multivariable-adjusted hazard ratios, with shaded areas showing 95% confidence intervals derived from restricted cubic spline regressions with four knots. Multivariate models were adjusted for baseline age, sex, marital status, education, residence, living arrangement, economic status, smoking status, drinking status, regular exercise, sleep time, body mass index, hypertension, heart disease, cerebrovascular disease, diabetes mellitus, respiratory disease, cancer, intake of fruit, intake of vegetable, intake of meat, intake of fish, and intake of egg.

**
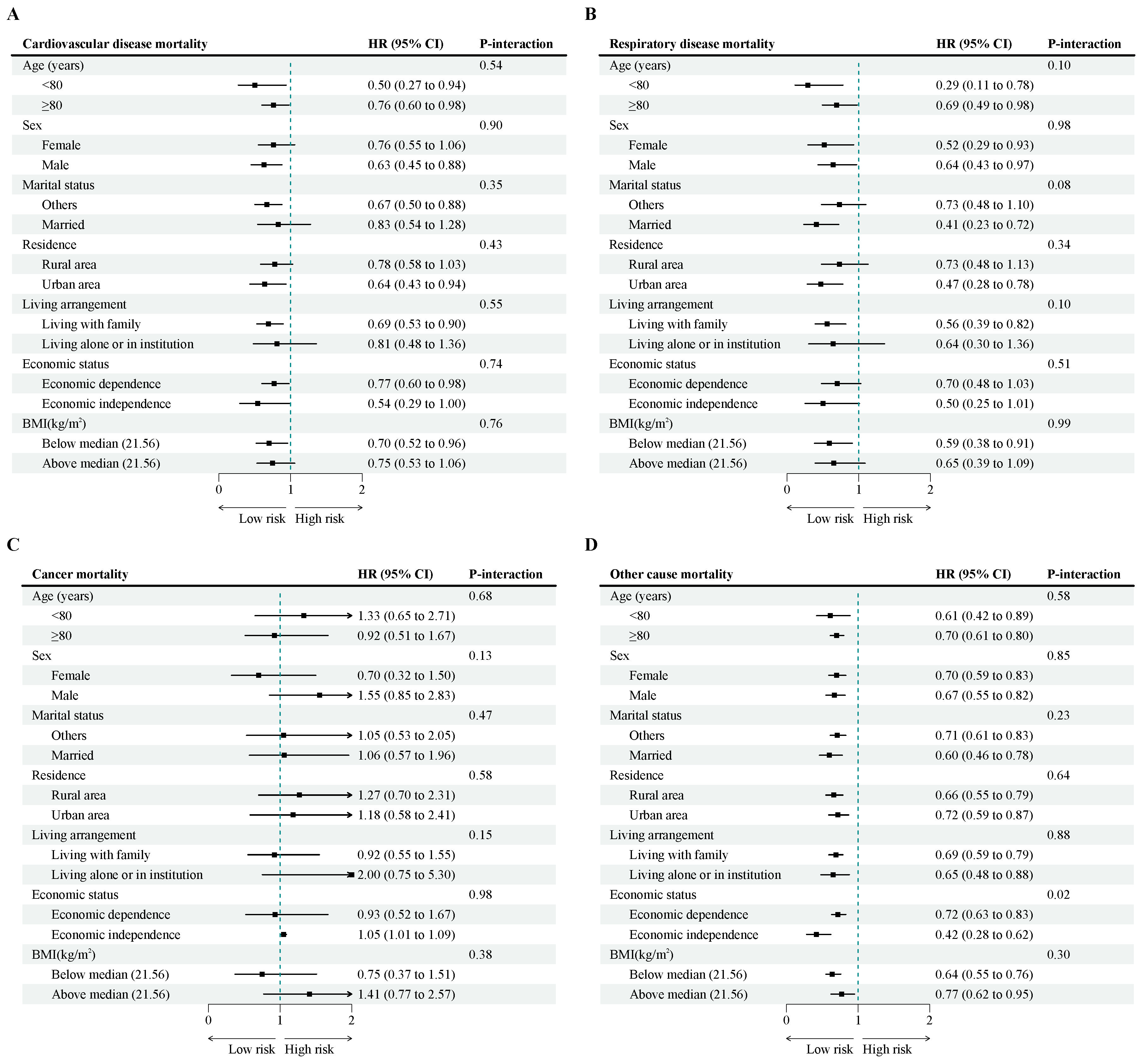
**

**Supplemental Figure 4** Association of psychological resilience with cause-specific mortality stratified by participant characteristics.

*HR* hazard ratio; *CI* confidence interval.

Notes: Each stratification controlled for all factors (baseline age, sex, marital status, education, residence, living arrangement, economic status, smoking status, drinking status, regular exercise, sleep time, body mass index, hypertension, heart disease, cerebrovascular disease, diabetes mellitus, respiratory disease, cancer, intake of fruit, intake of vegetable, intake of meat, intake of fish, and intake of egg) except the stratification factor itself.

**Supplemental table 1**

The coding of psychological resilience

| Items | Cut point |
| --- | --- |
| Feel the older you get, the more useless you are | 1=always; 2=often; 3=sometimes; 4=seldom; 5=never |
| Look on the bright side of things | 5=always; 4=often; 3=sometimes; 2=seldom; 1=never |
| Feel fearful or anxious | 1=always; 2=often; 3=sometimes; 4=seldom; 5=never |
| Feel lonely and isolated | 1=always; 2=often; 3=sometimes; 4=seldom; 5=never |
| Make own decisions concerning personal affairs | 5=always; 4=often; 3=sometimes; 2=seldom; 1=never |

**Supplemental table 2**

The numbers (percentage) of the missing variables

| Characteristics ^a^ | Number (%) with missing data |
| --- | --- |
| Marital status | 58 (1.2) |
| Living with family | 39 (0.8) |
| Education | 39 (0.8) |
| Economic status | 35 (0.7) |
| Smoking status | 22 (0.4) |
| Drinking status | 43 (0.9) |
| Regular exercise | 108 (2.2) |
| BMI | 281 (5.7) |
| Sleep time | 40 (0.8) |
| Intake of fruit | 9 (0.2) |
| Intake of vegetables | 8 (0.2) |
| Intake of meat | 43 (0.9) |
| Intake of fish | 44(0.9) |
| Intake of egg | 43 (0.9) |
| Hypertension | 275 (5.6) |
| Diabetes mellitus | 338 (6.8) |
| Heart disease | 313 (6.3) |
| Cerebrovascular disease | 315 (6.4) |
| Respiratory disease | 287 (5.8) |
| Cancer | 433 (8.8) |

^a^ List only the variables with missing data.

**Supplemental Table 3**

Demographic and clinical characteristics of the study population after propensity score matching

| Characteristics | Total (n = 3368) | Psychological resilience | | P value |
| --- | --- | --- | --- | --- |
|  |  | Low level (n = 1684) | High level (n = 1684) |  |
| Age (year), mean (SD) | 84.32 (10.05) | 84.33 (9.97) | 84.30 (10.13) | 0.93 |
| Female, no. (%) | 1794 (53.3) | 895 (53.1) | 899 (53.4) | 0.92 |
| Urban area, no. (%) | 1412 (41.9) | 690 (41.0) | 722 (42.9) | 0.28 |
| Married, no. (%) | 1414 (42.0) | 712 (42.3) | 702 (41.7) | 0.75 |
| living arrangement, no. (%) |  |  |  | 0.68 |
| Living with family | 2587 (76.8) | 1299 (77.1) | 1288 (76.5) |  |
| Living alone | 712 (21.1) | 348 (20.7) | 364 (21.6) |  |
| Living in institution | 69 (2.0) | 37 (2.2) | 32 (1.9) |  |
| Education (year), no. (%) |  |  |  | 0.87 |
| 0 | 1913 (56.8) | 956 (56.8) | 957 (56.8) |  |
| 1-6 | 1137 (33.8) | 573 (34.0) | 564 (33.5) |  |
| >6 | 318 (9.4) | 155 (9.2) | 163 (9.7) |  |
| Economic independence, no. (%) | 816 (24.2) | 403 (23.9) | 413 (24.5) | 0.72 |
| Smoking status, no. (%) |  |  |  | 0.64 |
| Never | 2374 (70.5) | 1195 (71.0) | 1179 (70.0) |  |
| Current | 597 (17.7) | 288 (17.1) | 309 (18.3) |  |
| Former | 397 (11.8) | 201 (11.9) | 196 (11.6) |  |
| Drinking status, no. (%) |  |  |  | 0.96 |
| Never | 2514 (74.6) | 1258 (74.7) | 1256 (74.6) |  |
| Current | 509 (15.1) | 252 (15.0) | 257 (15.3) |  |
| Former | 345 (10.2) | 174 (10.3) | 171 (10.2) |  |
| Regular exercise, no. (%) |  |  |  | 0.54 |
| Never | 2413 (71.6) | 1221 (72.5) | 1192 (70.8) |  |
| Current | 807 (24.0) | 392 (23.3) | 415 (24.6) |  |
| Former | 148 (4.4) | 71 (4.2) | 77 (4.6) |  |
| BMI (kg/m^2^), no. (%) |  |  |  | 0.93 |
| Underweight (<18.5) | 1912 (56.8) | 963 (57.2) | 949 (56.4) |  |
| Normal (18.5-24) | 564 (16.7) | 278 (16.5) | 286 (17.0) |  |
| Overweight (24-28) | 667 (19.8) | 334 (19.8) | 333 (19.8) |  |
| Obese (≥28) | 225 (6.7) | 109 (6.5) | 116 (6.9) |  |
| Sleep time (h), no. (%) |  |  |  | 0.97 |
| <6 | 545 (16.2) | 271 (16.1) | 274 (16.3) |  |
| 6-9 | 1915 (56.9) | 961 (57.1) | 954 (56.7) |  |
| ≥9 | 908 (27.0) | 452 (26.8) | 456 (27.1) |  |
| Intake of fruit, no. (%) |  |  |  | 0.92 |
| Daily | 417 (12.4) | 208 (12.4) | 209 (12.4) |  |
| Quite often | 972 (28.9) | 495 (29.4) | 477 (28.3) |  |
| Occasionally | 1193 (35.4) | 591 (35.1) | 602 (35.7) |  |
| Rarely or none | 786 (23.3) | 390 (23.2) | 396 (23.5) |  |
| Intake of vegetables, no. (%) |  |  |  | 0.69 |
| Daily | 1894 (56.2) | 941 (55.9) | 953 (56.6) |  |
| Quite often | 1132 (33.6) | 572 (34.0) | 560 (33.3) |  |
| Occasionally | 255 (7.6) | 132 (7.8) | 123 (7.3) |  |
| Rarely or none | 87 (2.6) | 39 (2.3) | 48 (2.9) |  |
| Intake of meat, no. (%) |  |  |  | 0.97 |
| Daily | 1274 (37.8) | 638 (37.9) | 636 (37.8) |  |
| Weekly | 1430 (42.5) | 708 (42.0) | 722 (42.9) |  |
| Monthly | 261 (7.7) | 131 (7.8) | 130 (7.7) |  |
| Occasionally | 184 (5.5) | 93 (5.5) | 91 (5.4) |  |
| Rarely or none | 219 (6.5) | 114 (6.8) | 105 (6.2) |  |
| Intake of fish, no. (%) |  |  |  | 0.98 |
| Daily | 270 (8.0) | 134 (8.0) | 136 (8.1) |  |
| Weekly | 1405 (41.7) | 708 (42.0) | 697 (41.4) |  |
| Monthly | 689 (20.5) | 337 (20.0) | 352 (20.9) |  |
| Occasionally | 465 (13.8) | 235 (14.0) | 230 (13.7) |  |
| Rarely or none | 539 (16.0) | 270 (16.0) | 269 (16.0) |  |
| Intake of egg, no. (%) |  |  |  |  |
| Daily | 949 (28.2) | 469 (27.9) | 480 (28.5) | 0.81 |
| Weekly | 1386 (41.2) | 705 (41.9) | 681 (40.4) |  |
| Monthly | 464 (13.8) | 228 (13.5) | 236 (14.0) |  |
| Occasionally | 251 (7.5) | 130 (7.7) | 121 (7.2) |  |
| Rarely or none | 318 (9.4) | 152 (9.0) | 166 (9.9) |  |
| Hypertension, no. (%) | 1156 (34.3) | 574 (34.1) | 582 (34.6) | 0.80 |
| Heart disease, no. (%) | 442 (13.1) | 224 (13.3) | 218 (12.9) | 0.80 |
| Diabetes mellitus, no. (%) | 195 (5.8) | 96 (5.7) | 99 (5.9) | 0.88 |
| Cerebrovascular disease, no. (%) | 295 (8.8) | 151 (9.0) | 144 (8.6) | 0.72 |
| Respiratory disease, no. (%) | 375 (11.1) | 184 (10.9) | 191 (11.3) | 0.74 |
| Cancer, no. (%) | 24 (0.7) | 10 (0.6) | 14 (0.8) | 0.54 |

*BMI* Body Mass Index.

Notes: Values were presented as number (%) or mean ± SD. Differences in characteristics were compared using the χ^2^ test for categorical variables and t-test for continuous variables.

**Supplemental Table 4**

Sensitivity analyses for the association of psychological resilience with all-cause and cause-specific mortality risk

| Characteristic | Removing participants with missing covariate data | Removing participants who had heart disease, diabetes mellitus, cerebrovascular disease, respiratory disease, or cancer | Removing the participants who died within the first year of follow-up | After propensity score matching |
| --- | --- | --- | --- | --- |
|  | HR (95% CI) | HR (95% CI) | HR (95% CI) | HR (95% CI) |
| All-cause mortality | | | |  |
| Low psychological resilience | Reference | Reference | Reference | Reference |
| High psychological resilience | 0.70 (0.62-0.80) | 0.70 (0.62-0.80) | 0.78 (0.69-0.88) | 0.71 (0.63-0.79) |
| Psychological resilience score | 0.93 (0.91-0.95) | 0.93 (0.91-0.95) | 0.94 (0.92-0.96) | 0.93 (0.91-0.95) |
| [Cardiovascular disease](javascript:;) mortality | | | |  |
| Low psychological resilience | Reference | Reference | Reference | Reference |
| High psychological resilience | 0.77 (0.58-1.03) | 0.80 (0.59-1.09) | 0.82 (0.63-1.07) | 0.69 (0.53-0.89) |
| Psychological resilience score | 0.93 (0.89-0.98) | 0.95 (0.90-1.00) | 0.95 (0.91-0.99) | 0.92 (0.88-0.96) |
| Respiratory disease mortality | | | |  |
| Low psychological resilience | Reference | Reference | Reference | Reference |
| High psychological resilience | 0.55 (0.38-0.82) | 0.38 (0.24-0.61) | 0.63 (0.43-0.93) | 0.52 (0.36-0.75) |
| Psychological resilience score | 0.88 (0.82-0.94) | 0.85 (0.80-0.91) | 0.91 (0.85-0.97) | 0.89 (0.84-0.95) |
| Cancer mortality | | | |  |
| Low psychological resilience | Reference | Reference | Reference | Reference |
| High psychological resilience | 1.22 (0.73-2.04) | 1.20 (0.67-2.12) | 1.00 (0.60-1.66) | 0.86 (0.51-1.45) |
| Psychological resilience score | 0.98 (0.90-1.07) | 0.96 (0.87-1.05) | 0.97 (0.89-1.05) | 0.94 (0.86-1.03) |
| Other cause mortality | | | |  |
| Low psychological resilience | Reference | Reference | Reference | Reference |
| High psychological resilience | 0.62 (0.54-0.73) | 0.66 (0.56-0.77) | 0.73 (0.63-0.85) | 0.66 (0.57-0.76) |
| Psychological resilience score | 0.91 (0.89-0.93) | 0.92 (0.89-0.94) | 0.93 (0.91-0.95) | 0.91 (0.89-0.94) |

*HR* hazard ratio; *CI* confidence interval.

Notes: Multivariate models were adjusted for baseline age, sex, marital status, education, residence, living arrangement, economic status, smoking status, drinking status, regular exercise, sleep time, body mass index, hypertension, heart disease, cerebrovascular disease, diabetes mellitus, respiratory disease, cancer, intake of fruit, intake of vegetable, intake of meat, intake of fish, and intake of egg.
